# Prevalence and Factors Associated with the Use of Artificial Sweeteners in Nonpregnant, Nonlactating Females of Reproductive Age – A Systematic Review

**DOI:** 10.1016/j.cdnut.2025.107478

**Published:** 2025-06-04

**Authors:** Saima Shaukat Ali, Patience Elizabeth Castleton, Kainat Meherali, Mumtaz Begum, Shao Jia Zhou, Zohra S Lassi

**Affiliations:** 1 Robinson Research Institute, University of Adelaide, Australia; 2 School of Public Health, Faculty of Health and Medical Sciences, University of Adelaide, Australia; 3 Department of Pharmacology, Faculty of Science, University of Alberta, Canada; 4 Adelaide Medical School, Faculty of Health and Medical Sciences, University of Adelaide, Australia; 5 Department of Food and Nutrition, School of Agriculture, Food and Wine, University of Adelaide, Australia

**Keywords:** artificial sweetener (AS), nonnutritive sweetener (NNS), nonpregnant nonlactating females, females of reproductive age, women of reproductive age

## Abstract

**Background:**

Artificial or nonnutritive sweeteners (ASs/NNSs) are widely consumed as sugar substitutes globally. Although research often focuses on specific groups such as those with diabetes or pregnant females, nonpregnant, nonlactating females of reproductive age remain understudied despite the significant implications of dietary patterns on females’ health.

**Objectives:**

This systematic review aims *1*) to summarize the global prevalence of AS/NNS consumption among nonpregnant, nonlactating females aged 15−49, and *2*) to identify the factors associated with AS/NNS use within this population.

**Methods:**

Following PRISMA reporting guidelines, we conducted a comprehensive search across multiple databases, including the Cochrane Database of Systematic Reviews, CENTRAL, Campbell Library, MEDLINE, EMBASE, CINAHL, SCOPUS, Web of Science, and eLANA (World Health Organization), covering studies up to May 2024. We included observational and experimental studies involving nonpregnant and nonlactating females aged 15 to 49, focusing on AS/NNS consumption and its associated factors. Data extraction was performed in duplicates, and quality assessment was conducted using the National Heart, Lung, and Blood Institute Quality Assessment Tool.

**Results:**

This review identified 15 eligible studies. The pooled prevalence of AS/NNS use among females of reproductive age was 37.1% (95% confidence interval: 25.1%, 49.9%; heterogeneity: *I*^2^ = 99.9%). Most of the studies were conducted in high-income countries, with only one from an upper-middle-income country. All studies were cross-sectional (*n* = 15), with 33% high and 67% moderate quality ratings. Factors associated with AS/NNS use included people living with obesity, ethnicity (higher prevalence among White participants), and physical activity levels. However, due to a lack of sex-disaggregated data, these factors could not be analyzed specifically for females; thus, a narrative synthesis is presented.

**Conclusion:**

This review highlights significant AS/NNS consumption among nonpregnant, nonlactating females of reproductive age. The findings underscore the need for further research on the long-term health implications of AS/NNS use in this population.

The study was registered with PROSPERO as CRD42023450145.

## Introduction

Artificial sweeteners (ASs), or nonnutritive sweeteners (NNSs), are synthetically produced compounds used as low-calorie substitutes for sugar [1]. Recent decades have seen a notable shift in consumer preferences to these advertised “healthier” products, thus increasing the commercial use of ASs/NNSs in a wide range of products and promoting their popularity in household use [2].

Diverse global guidelines exist regarding AS/NNS use and sales depending on each country’s food regulation rules. For example, the United States Food and Drug Administration (FDA) has approved 5 ASs/NNSs (acesulfame-K, aspartame, advantame, neotame, saccharin, and sucralose) with 2 others (steviol glycosides and Luo Han Guo fruit [monk fruit extract]) regarded as safe and not needing FDA approval [3]. However, in addition to those approved by the FDA, the additional ASs/NNSs alitame, aspartame-acesulfame salt, cyclamate, and thaumatin have also received approval from the Food Standards Australia and New Zealand [4].

The rising global obesity epidemic, and its accompanying adverse health outcomes, has been linked to high sugar consumption [[5], [6], [7]], thus prompting the WHO to recommend limiting sugar consumption to 10% of total daily energy intake [8]. As such, there has been an increase in AS/NNS purchasing and consumption globally [9,10] with variations in trends and consumption patterns in different countries. For example, in the United States, low-calorie sweetener (LCS) beverage consumption increased from ∼27% to 32% between 1999−2000 and 2007−2008 [11]. The study reported that the most significant group to increase use of LCS was females [11]. AS/NNS promotions portray the products as low/no-calorie “healthy” alternatives to sugar, and despite the increase in their consumption, rising obesity prevalence, related health issues, and comorbidities remain persistently on the rise globally [6,12].

Literature on the health implications of AS/NNS use is conflicting, with some studies showing links to increased risk of metabolic syndrome [13], diabetes mellitus [14], cardiovascular problems [15], depression [16], dementia [17], osteoporosis [18], and some types of cancer [19], whereas others show the positive impacts of AS/NNS substitution on weight loss/maintenance [20]. Consumption of ASs/NNSs during pregnancy has also been linked with neonatal outcomes, including preterm delivery and increased birth weight [21], and the recent WHO guidelines discourage its use for weight control [12]. Therefore, it is important to judge the quality of the published studies to understand the full extent of AS/NNS consumption among this population and disentangle the factors associated with their use.

Females are among the highest consumers of ASs/NNSs [22,23], often motivated by body dissatisfaction and weight loss [24]. However, nonpregnant and nonlactating females of reproductive age have unique dietary patterns and health considerations that are frequently overlooked in this research. Moreover, the available literature often aggregates diverse age groups, making it difficult to discern consumption trends and associated factors specific to this critical life stage.

The focus of this systematic review on nonpregnant and nonlactating females aged 15 to 49 y is grounded in both scientific and demographic considerations. Females within this age range experience unique physiological and metabolic conditions influenced by hormonal fluctuations, reproductive health, and lifestyle factors. Research often fails to disaggregate data specific to this group, leading to a lack of granularity in understanding their dietary patterns and associated factors and health outcomes. Additionally, this life stage encompasses critical health and social dynamics for which dietary decisions, such as AS/NNS use, could significantly impact metabolic health, hormonal balance, and mental health [25,26]. By excluding pregnant and lactating females, this study mitigates the effect of changes introduced by pregnancy-specific dietary needs and hormonal alterations, thereby enabling a clearer understanding of AS/NNS consumption patterns and associated factors.

Considering these gaps, this systematic review aimed to consolidate global evidence on the prevalence and patterns of AS/NNS consumption among females of reproductive age while identifying the factors influencing their use. By focusing on this understudied demographic, the review intends to address a significant void in nutritional research, equipping health care providers and policymakers with insights to guide education and decision making. Ultimately, this work aspires to empower females to make informed dietary choices during this pivotal life stage, fostering improved health outcomes and addressing critical knowledge gaps.

The objectives of this study were *1*) to estimate the pooled global prevalence of AS/NNS consumption in nonpregnant, nonlactating females aged between 15 and 49 y, and *2*) to identify the factors globally associated with AS/NNS consumption among nonpregnant, nonlactating females aged between 15 and 49 y.

## Methods

We followed the PRISMA [27] guidelines for reporting this systematic review and registered the protocol with PROSPERO (CRD42023450145).

We included studies conducted from inception until May 2024 that involved nonpregnant females aged 15 to 49 years residing in any global setting who consumed ASs/NNSs, according to the inclusion criteria detailed in Table 1. A search strategy was constructed using the population, intervention, comparison, and outcome criteria. Major themes and keywords related to our study were used as search terms for each database search, as detailed in Supplemental Appendix 1. We then systematically searched the following scientific databases for relevant studies: Cochrane Database of Systematic Reviews and the Cochrane Central Register of Controlled Trials in the Cochrane Library, the Campbell Library, MEDLINE (PubMed), EMBASE, CINAHL, SCOPUS, Web of Science, and eLANA (WHO). In addition to these databases, the reference lists of all the articles included in this review were screened to identify further potential articles. Initial searches were conducted on 24 April, 2023, and then revised on 9 May, 2024.

**TABLE 1:** Inclusion and exclusion criteria of studies included in the systematic review.

	Included	Excluded
Population	Childbearing/reproductive age females aged 15–49 y.	Studies describing females aged <15 or >49 y or do not explicitly mention the age of participants. Studies focused on pregnant/lactating females, males, nondisclosed genders, or other genders.
Study design	Observational studies (prospective and retrospective cohort studies and cross-sectional studies), experimental [randomized (individually or cluster)], and nonrandomized controlled trials including quasi-randomized trials and controlled before-after studies. Studies published in English until 2024.	Animal studies, studies that are not in the English language, systematic and narrative reviews, case reports, case studies, opinions, editorials, commentaries, and letters.
Exposure/intervention	Global studies that assessed the consumption and associated factors of one/more/combination of ASs saccharin, aspartame, acesulfame-K, sucralose, neotame, advantame, and xylitol consumed through all dietary sources (food or beverages).	No exclusions applied.
Outcome	Global studies that examined the proportion of use of ASs (overall as well as based on types of AS) in females aged 15–49 y. Global studies that examined associated factors of AS use in females aged 15–49 y (overall as well as based on types of AS) such as but not limited to socioeconomic status, age, marital status, lifestyle, and health conditions.	No exclusions applied.
Setting	Studies from any setting (urban and rural areas in low-, middle-, and high-income countries) are included.	No exclusions applied.

AS, artificial sweetener.

**FIGURE 1:**
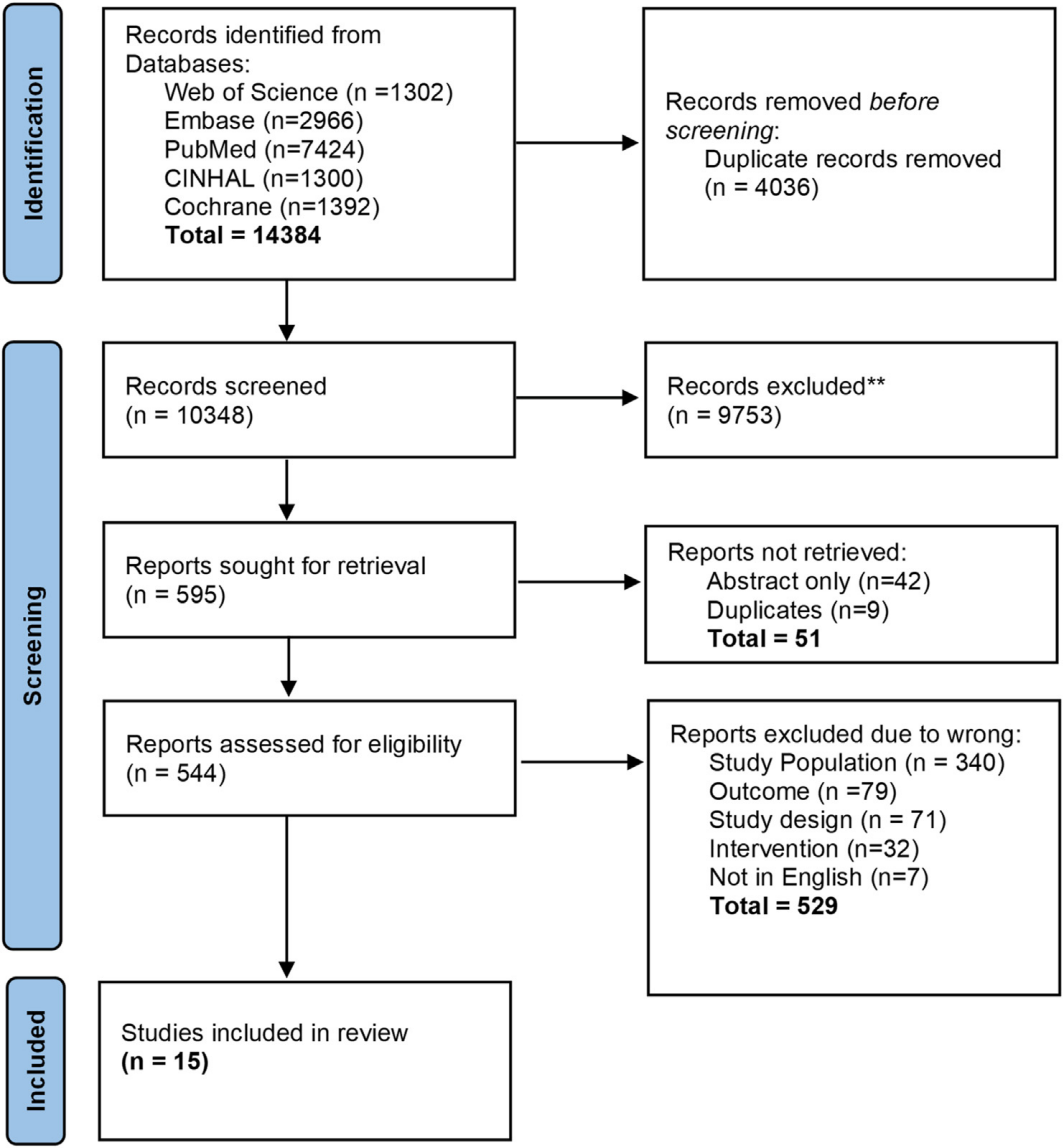
PRISMA flowchart.

Search results were exported into EndNote, deduplicated, and uploaded into Covidence. Three authors (SSA, KM, and PC) independently screened the titles and abstracts of all studies in duplicate. After title and abstract screening, full texts were independently screened for inclusion by 3 review authors (SSA, KM, and PC) in duplicate. Any disagreements were resolved through discussion or by consulting a third author (ZL) if required. Reasons for exclusion were recorded for all the studies excluded at the stage of full-text screening and are reported in Figure 1.

Two review authors (KM and PC) performed data extraction, independently extracting data on an Excel spreadsheet developed by the first author (SSA), including information on the publication year, study design, sample size, population age, and demographics. Included studies were independently extracted twice for data related to the prevalence of AS/NNS use in the study population, including information on the sources of consumed sweeteners, the frequency of consumption, and the factors associated with their use, such as education levels and socioeconomic status.

Two authors (PC and SSA) independently assessed the quality of all included studies using the National Heart, Lung, and Blood Institute (NHLBI) Quality Assessment Tool for Studies [28]. Discrepancies in evaluations were resolved by discussions with a third author (ZL).

Using JBI SUMARI software [29], we assessed the pooled prevalence of AS/NNS use using the Freeman-Tukey transformation. Due to high heterogeneity among the included studies, we used a random-effects model. We planned to analyze the factors associated with AS/NNS use compared to nonuse of ASs/NNSs in the included studies using the Cochrane Review Manager. However, each of the included studies presented these data in different ways, not aggregating their results by sex or by age; thus, we could not meta-analyze the data. Therefore, we chose to narratively report those findings. However, we presented the results based on the mode of consumption (i.e., in beverage or food form) and also conducted a sensitivity analysis for studies with high methodological quality.

## Results

**FIGURE 2:**
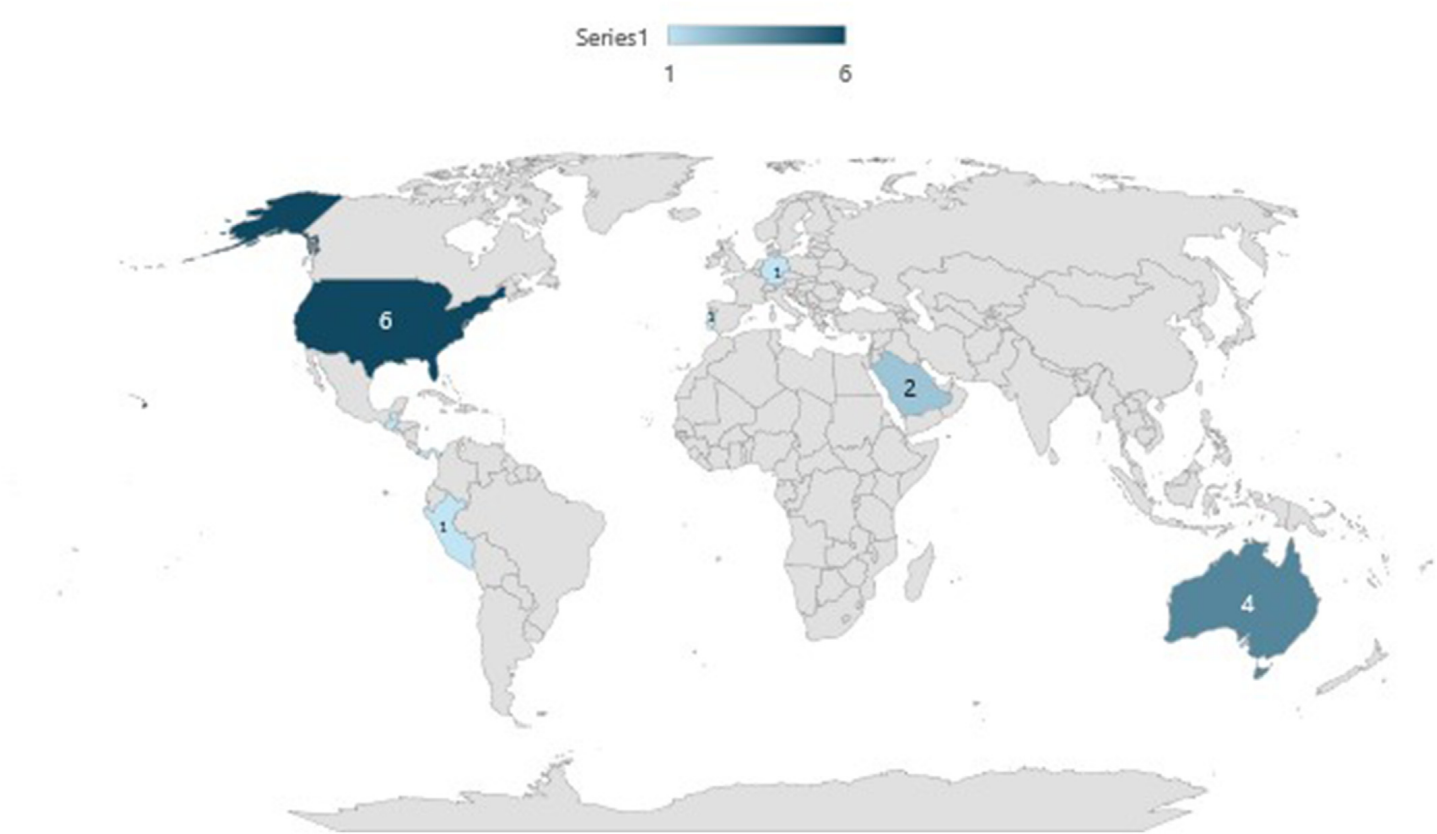
Heat map showing the country distribution of included studies.

As depicted in the PRISMA flow diagram (Figure 1), the search yielded 14,384 results. After screening titles and abstracts, 544 studies were assessed at the full-text stage, and 15 studies met the eligibility criteria and were included in the final review [11,[30], [31], [32], [33], [34], [35], [36], [37], [38], [39], [40], [41], [42], [43]]. All 15 studies included in this review were cross-sectional studies, with 6 being conducted in the United States [11,35,38,39,41,43], 4 in Australia [32,36,37,43], 2 in Saudi Arabia [31,40], and 1 each in Portugal [33], Germany [30], and Latin America (Peru, Chile, Guatemala and Panama) [34] (Figure 2).

Although 11 studies [11,[30], [31], [32], [33],[37], [38], [39],[41], [42], [43]] involved the general population, 2 studies each recruited university students only [34,40] and high school adolescents [35,36]. Of all the studies, only 1 study from Australia included female participants from the general population [32]. The remaining 14 included both male and female participants [11,30,31,[33], [34], [35], [36], [37], [38], [39], [40], [41], [42], [43]]. For the first aim, which was to assess prevalence, we included only the prevalence of AS use among nonpregnant, nonlactating females. However, for the second objective, the available data were mixed for males and females; therefore, we noted this as a limitation and did not perform a pooled analysis.

Three studies specified the sweeteners acesulfame, aspartame, cyclamate, and saccharin were consumed [30,33,34], whereas 2 reported sucralose [33,34] and 1 reported steviol glycosides [33] were consumed. Nine of the included studies measured ASs/NNSs in both food and drinks, whereas the rest of the 5 studies measured ASs/NNSs in beverages, with 1 study further specifying AS/NNS added in hot beverages [40].

**FIGURE 3:**
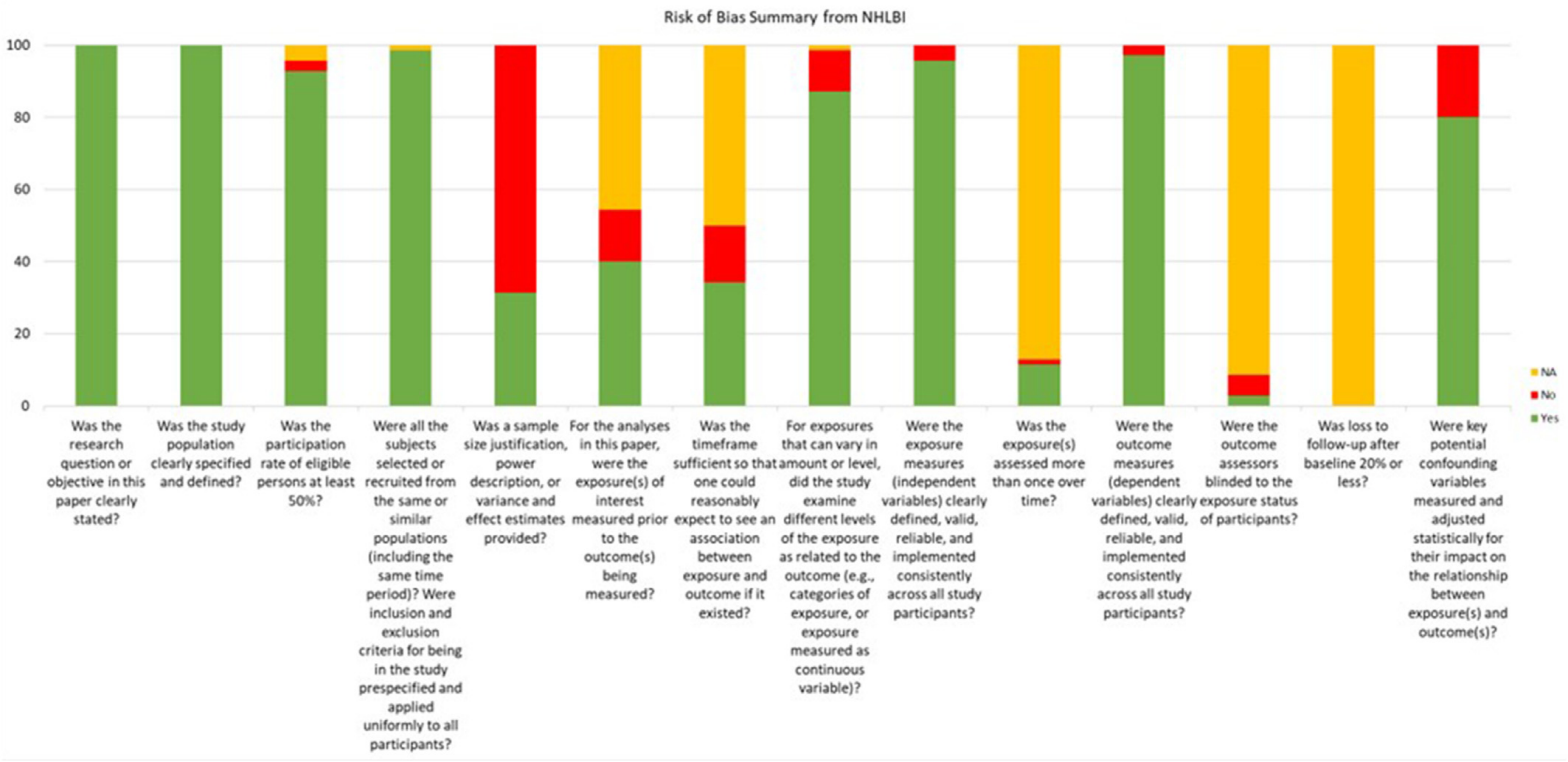
Quality assessment of the included studies based on National Heart, Lung, and Blood Institute criteria.

Additionally, 4 studies completed secondary analysis of NHANES data. Of these, Shoham et al. [43] reported on 1999−2004 data, Malek et al. [41] reported on 2007−2012 data, Sylvetsky et al. [11] reported on 1999−2000 and 2007−2008 data, and Leahy et al. [39] reported on 2001−2012 data. Grech et al. [37] analyzed data from the 2011/2012 Australian National Nutrition and Physical Activity Survey, and Pollard et al. [42] used the Western Australia Department of Health’s Nutrition Monitor Survey Series data. Leahy et al. [39] reported on data collected from NHANES 2001−2012; however, this data set overlaps with data collected by Malek et al. [41] and Shoham et al. [43], who collected data from 2007−2012 and 1999−2004, respectively. Therefore, we excluded Leahy et al. [39] from the pooled analysis to prevent the skewing of results from overlapping datasets. Thus, the pooled prevalence comes from 14 studies, as shown in Figure 3.

The included studies in this systematic review exhibited varying levels of quality, as assessed using the NHLBI quality assessment tool. Among the 15 studies evaluated, 5 were rated as high quality [[31], [32], [33],^36^,^40^], indicating robust methodologies and minimal risk of bias. The remaining 10 studies were assessed as moderate quality, reflecting sound research practices but with some limitations that may affect the strength of their findings (Figure 3).

High-quality studies had minimal risk across domains [[31], [32], [33],^36^,^40^], making their findings highly reliable. The remaining studies showed some limitations, particularly in sample size justification, measurement reliability, and control of confounding factors. However, these issues do not significantly undermine their contributions. The table in Supplemental Appendix 2 details risk of bias assessment.

### Prevalence of AS/NNS use

**FIGURE 4:**
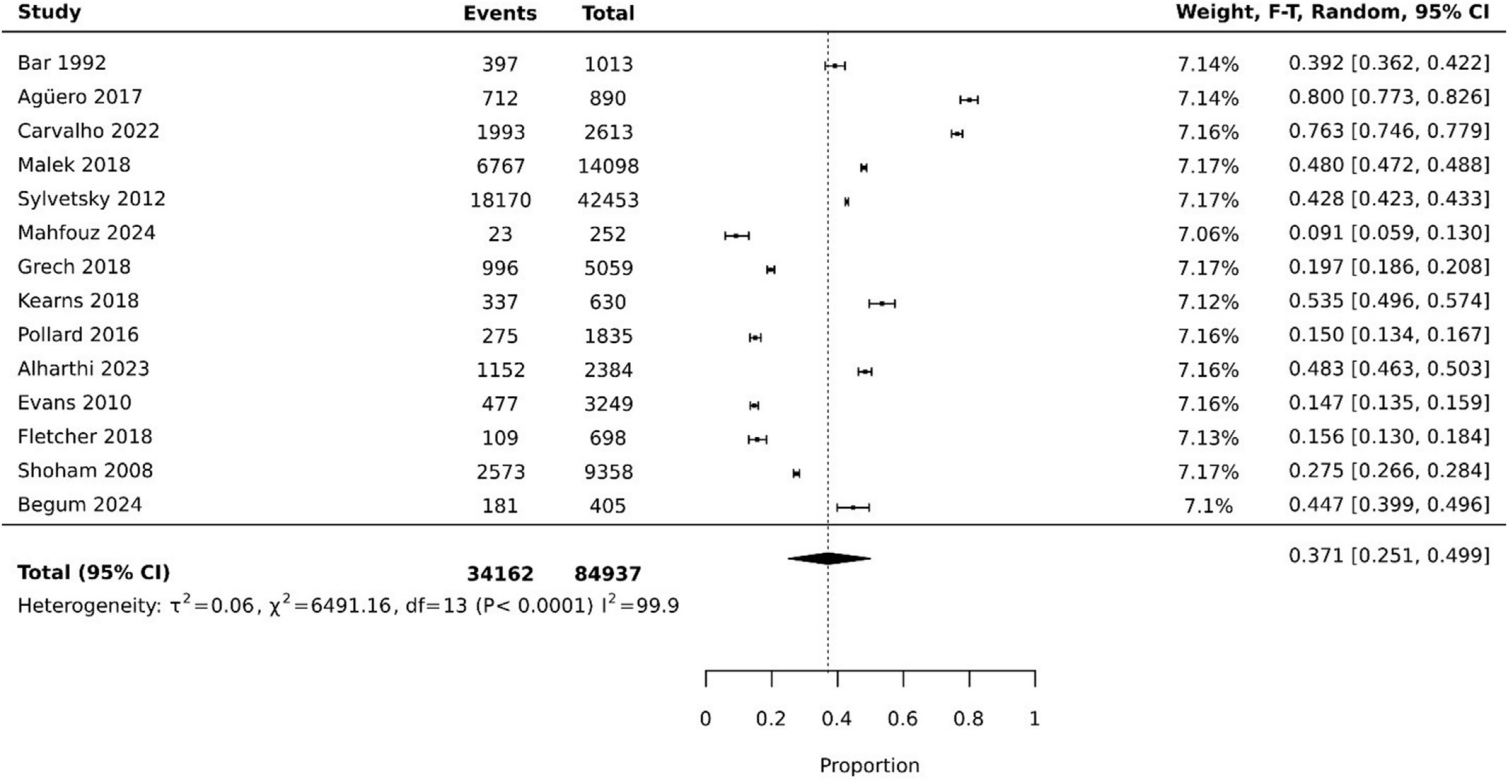
Pooled prevalence of artificial sweetener/nonnutritive sweetener consumption among nonpregnant and nonlactating females of reproductive age. CI, confidence interval, F-T, Freeman-Tukey.

All included studies reported on the prevalence of AS/NNS use among females of reproductive age with a pooled prevalence of 37.1% [95% confidence interval (CI): 25.1%, 49.9%; heterogeneity: *I*^2^ = 99.9%; random effect model] (Figure 4). The minimum recorded AS/NNS consumption prevalence in the 14 pooled studies was 9.1% [40], with the maximum prevalence being 80% [34].

**FIGURE 5:**
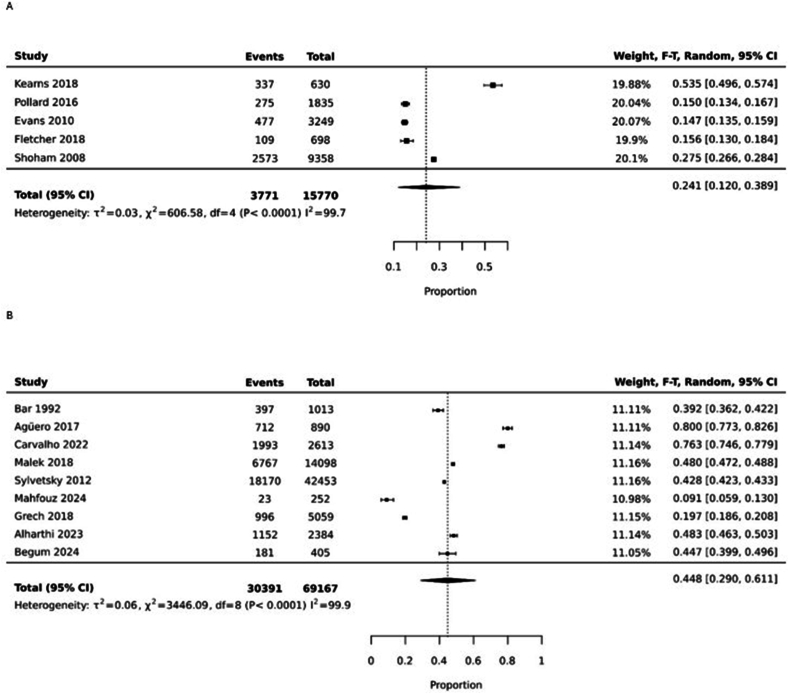
(A) Pooled prevalence of the source of artificial sweeteners/nonnutritive sweeteners as beverages only. (B) Pooled prevalence of the source of artificial sweeteners/nonnutritive sweeteners as both food and beverage. CI, confidence interval, F-T, Freeman-Tukey.

We performed subgroup analysis on the source of ASs/NNSs as beverage only or as both food and beverage. The pooled prevalence of AS/NNS consumption as a beverage was 24.1% (95% CI: 12%, 38.9%; heterogeneity: *I*^2^ = 99.7%; random effect model) and as both food and beverage was 44.8% (95% CI: 29.0%, 61.1%; heterogeneity: *I*^2^ = 99.9%; random effect model) (Figure 5A, B).

**FIGURE 6:**
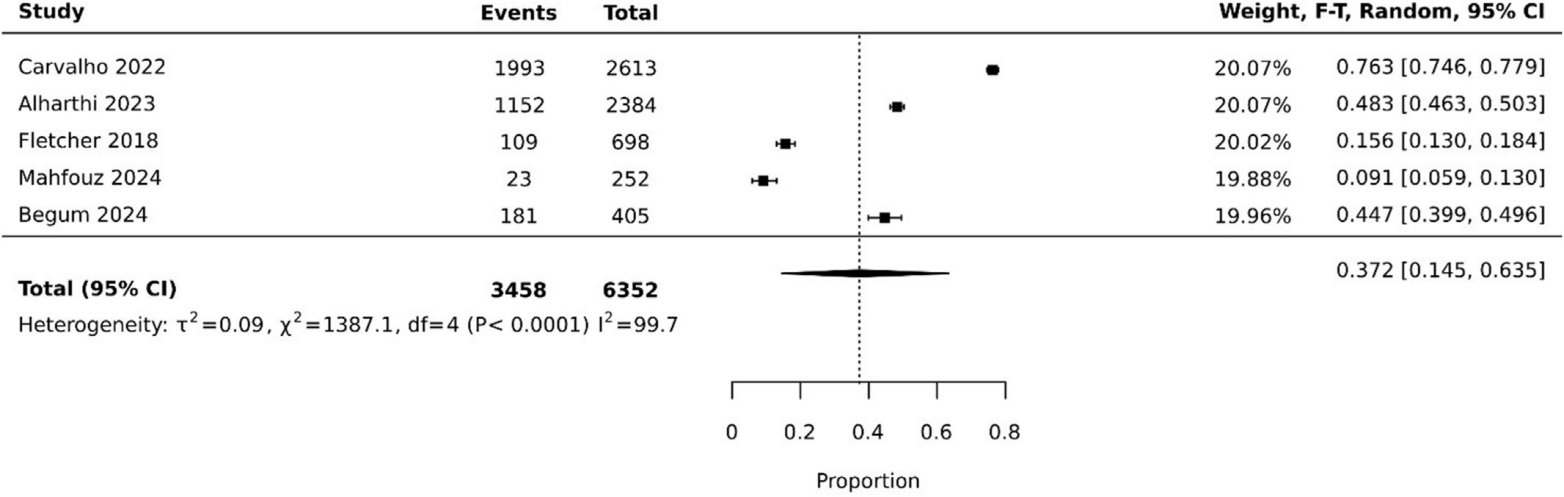
Pooled prevalence of artificial sweetener/nonnutritive sweetener consumption among nonpregnant and nonlactating females of reproductive age (high methodological quality studies only). CI, confidence interval, F-T, Freeman-Tukey.

For sensitivity analysis, we separated studies based on methodological quality, and pooled prevalence was 37.2% (95% CI: 14.5%, 63.5%; heterogeneity: I^2^=99.7%) (Figure 6).

### Analysis of factors associated with AS/NSS use

Ten of the included studies discussed different factors associated with AS/NSS intake in their populations. However, only 1 study explicitly investigated the factors associated with AS/NNS use among nonpregnant, nonlactating females of reproductive age [32]. It is important to note that the majority of the studies included both males and females in their populations; hence, the following discussions on the factors associated with AS/NNS consumption are not female-specific and are instead based on the entire study populations.

Seven studies assessed the associations between BMI (in kg/m^2^) and AS/NNS usage [37,39,40,44]. Five studies found that obesity was associated with a higher prevalence of AS/NNS usage than normal weight [37,39,40,44], whereas 3 found the opposite result [31,33,40]. Two of these studies [31,33] did show that, although individuals of “normal” weight reported a higher intake of ASs/NNSs than obese individuals, obese individuals had higher AS/NNS intake (30.77% [31], 36.8% [33]) than overweight individuals (27.71% [31], 36.7% [33]). Additionally, Leahy et al. [39] reported on those who regularly participated in vigorous exercise consumed more no- and low-calorie sweetened beverages (39.5%) compared to those who participated in moderate (34.6%) and sedentary activities (25.9%); however, the authors did not comment on the BMI of participants.

Six studies analyzed the association of ethnicity (African American, Mexican American, Hispanic, non-Hispanic White, non-Hispanic Black, Aboriginal/Torres Strait Islander, others) with AS/NNS use and found that White participants had the highest prevalence of AS/NNS use [35,38,39,41,44], and 1 study reported 71.3% of White participants consumed low and low-calorie beverages [39]. Further, Black participants had the lowest reported prevalence in all studies (34.4% [41], 22.4% [44], 10.9% [39], 12.6% [35], and 7.2% [38]).

Individuals with higher socioeconomic position and/or income had the highest prevalence of AS/NNS use compared with those with low socioeconomic position in 4 studies that reported on participant income [35,39,41,44]. However, Grech et al. [37] reported the highest AS/NNS usage among those with low socioeconomic position (19.4%) compared to medium (18.1%) and high status (17.6%). Leahy et al. [39] also noted that those in the lowest poverty index ratio (<1.3%) had a higher prevalence of AS/NNS use compared to those in the middle poverty index ratio (1.3% < poverty index ratio < 1.85%) (21.5% compared with 10.1%). Evans et al. [35] reported similar findings that those at medium disadvantage had the highest prevalence of AS/NNS use (14.4%) compared to those at low disadvantage (17.5%) and high disadvantage (12.8%). One study [32] reported that females of reproductive age (nonpregnant and nonbreastfeeding) who participated in a weight loss program, who had high BMIs, and who were using LCSs for weight loss reasons were more likely to be moderate or heavy LCS users than light users. In addition, socioeconomically disadvantaged females (those with lower education, who were divorced/widowed, or who were retired or unable to work) were less likely to be moderate or heavy LCS users [32].

Six studies reported on associations between education level and AS/NNS intake [31,33,37,38,41]. Five [31,33,37,38,41] of these reported higher prevalence with higher education, whereas 1 [32] reported that females of reproductive age with lower education were less likely to be moderate or heavy LCS users. Alharthi et al. [31] reported that high school graduates were most likely to use ASs/NNSs compared to those currently in school (88.67% compared with 8.49%) and those who were illiterate (2.83%). These findings were verified by Kearns et al. [38] and Malek et al. [41], both reporting the highest AS/NNS prevalence among college graduates (38.5% and 58%, respectively). Carvalho et al. [33] similarly reported highest AS/NNS prevalence among those with >12 y of education (43.6%), compared to those with 7 to 12 y of education (40.7%) and <6 y of education (29.1%). However, Grech et al. [37] found that, although college graduates had the highest reported use of ASs/NNSs compared to vocational college participants (18.6% vs. 18.0%), participants with high school education or less had a slightly higher prevalence of AS/NNS use (18.3%) compared to those with a vocational college education. One study reported that a high proportion of females did not consume the recommended servings of vegetables (57.8%), dairy (44.2%), meat (48.2%), and grains (74.8%) [32].

In summary, most studies reported high AS/NNS consumption among socioeconomically advantaged populations. Additionally, 3 studies [30,33,35] commented on associations between smoking and AS/NNS usage, all interestingly finding that nonsmokers consumed more ASs/NNSs than smokers.

## Discussion

This systematic review of 15 cross-sectional studies on AS/NNS consumption among females of reproductive age reveals notable patterns in prevalence and its associated factors. The prevalence of AS/NNS use was estimated at 37.1%, with a high level of heterogeneity across studies (*I*^2^: 99.9%), indicating substantial variability in study methods, population characteristics, or both. Despite the use of sensitivity analysis, heterogeneity remained high, preventing meaningful subgroup analyses, although factors such as type of ASs/NNSs, age range, and form of consumption were largely consistent across studies.

The included studies were from 9 different countries (Peru, Chile, Guatemala, Panama, Portugal, United States, Saudi Arabia, Australia, and Germany), providing an in-depth overview of the use of ASs/NNSs among females of reproductive age worldwide. We were able to identify factors related to high AS/NNS use, including high BMI, White ethnicity, high economic status, high education level, and nonsmoking status. However, only one of the included studies reported on these factors separately for females only [32]. This study identified education level, participation in weight management activities, and chronic health conditions such as diabetes as prominent factors associated with AS use [32]. Behavioral patterns, such as frequent consumption of sugar-free products and higher physical activity levels, were also strongly linked to AS/NNS consumption. These findings suggest that AS/NNS use is multifaceted, influenced by a combination of socioeconomic, health, and lifestyle factors. Notably, knowledge and perception about ASs/NNSs emerged as a critical determinant of usage, with those who perceive ASs/NNSs as beneficial demonstrating higher consumption. However, concerns about potential health risks, including metabolic disorders, also shaped the decision-making process for some participants. The identification of these predictors provides valuable insights into the nuanced behaviors driving AS consumption in females of reproductive age. However, the generalizability of these findings is limited, except for one study that was conducted among females in Australia [32]. The remaining studies discussed included both male and female participants of all ages.

Income and education levels were positively associated with AS/NNS consumption in most studies, suggesting that individuals with higher socioeconomic positions are more likely to use these products. Two of the included studies [33,41] found that higher-income participants had a greater prevalence of AS/NNS use, which could be related to greater access to a variety of low-calorie products and possibly more awareness of dietary options. Interestingly, an Australian study using 2011−2012 data reported no significant associations between socioeconomic status or education with the use of ASs/NNSs; however, these were crude estimates [37]. A recent study using 2023 data from Australia identified that females with lower education levels had lower consumption rates [32]. This discrepancy may reflect differences in confounder adjustment or changes over time in food accessibility. A recent global review showed that, although the highest global use of ASs/NNSs was among high-income countries (HICs), the use of ASs/NNSs in upper-middle- and lower-middle-income countries (LMICs) is continually increasing [45], which may also help to explain the discrepancy in study findings.

Three included studies reported that individuals with high levels of education, particularly those with college degrees, were more likely to consume ASs/NNSs [33,37,41]. This finding is confounding in some of the other included studies and in other current literature that indicates that those with lower education levels are more likely to consume ASs/NNSs [38]. One possible explanation for this discrepancy is the study location of the research, as college graduates in HICs may be more exposed to health-related information, advertisements, dietary trends, and social media advertisements of ASs/NNSs.

In HICs, individuals with higher education levels are more likely to engage with health campaigns and make health-conscious dietary decisions [46], such as choosing low-calorie sweeteners to reduce sugar intake [32,47]. Furthermore, higher education often provides greater access to information and products related to health [48], leading to a decreased intake of sugar-sweetened beverages in some studies [49,50]. However, this could, in turn, lead to more awareness and use of alternatives such as ASs. This could explain why, in our study, college graduates in these settings may consume ASs more frequently than their less-educated counterparts.

Ethnicity emerged as a significant factor in AS/NNS use, with White participants having the highest prevalence across multiple studies [11,38,41]. Conversely, Black participants reported the lowest AS/NNS use in multiple other studies, highlighting potential cultural and socioeconomic disparities in dietary behaviors [9,51]. A recent study conducted in Australia showed highest consumption among females of South Asian origin and Aboriginal Australians [32]. A study conducted in 2014 found that White American households had a higher percentage of purchasing low-calorie drinks as compared to African American and Hispanic households [9]. This heavily indicates that cultural attitudes toward ASs, historical dietary patterns, and access to dietary information are likely to influence AS/NNS consumption. The consistent trend across different studies suggests that ethnicity must be considered in public health messaging and interventions related to AS/NNS use.

The association between AS/NNS use and BMI was somewhat complex. Seven studies assessed the relationship, with 2 finding that obesity was associated with higher AS/NNS consumption [34,38]. However, 2 studies noted that normal-weight individuals consumed more ASs/NNSs than overweight or obese participants, although obese individuals generally had higher AS/NNS intake compared to those who were overweight [31,33]. Leahy et al. [39] further found that participants who engaged in vigorous physical activity had a higher prevalence of AS/NNS use, potentially indicating that individuals focusing on fitness or weight control may incorporate ASs/NNSs as part of their dietary routines. Studies have shown that a major reason for AS/NNS use is for weight loss and healthy lifestyle purposes [52,53]. Therefore, motivations to consume ASs/NNSs may differ between BMI groups; for example, those of normal weight may use ASs/NNSs for dietary flexibility, whereas those with higher BMI may use it for weight management purposes. However, recent studies have shown no significant or beneficial effects of ASs/NNSs in weight loss in children and adults [54,55]. In addition, the Dietary Guidelines for Americans (2015) indicate that LCS may be useful for short-term weight management, but the long-term effectiveness of LCSs remains unclear [56]. In 2023, the WHO released new guidelines advising against the use of LCSs for weight control, citing potential undesirable health implications from long-term consumption [12] thus indicating that widespread education and awareness around the use of ASs/NNSs is required.

An interesting observation from included studies [31,38] was that nonsmokers reported higher AS/NNS consumption than smokers. This finding is supported by previous studies in China [57] and the United States [58] that found positive relationships between and sugar-sweetened soda and tobacco consumption. Interestingly, a recent meta-analysis showed significantly lower health literacy among smokers than nonsmokers [59]. This may explain the relationship between smoking and higher AS/NNS consumption; however, further investigation is needed to better understand the behavioral links between smoking and AS/NNS use.

A similar systematic review and meta-analysis assessing AS/NNS use and its association with type 2 diabetes risk in the United States and UK reported a relative risk for artificial sweetened beverages of 1.48, prior to adjusting for confounders [60], supporting the results of the present study. However, these results were not aggregated by sex or age, and thus this prevalence may be slightly lower for the females of reproductive age involved. Interestingly, the United States/UK meta-analysis found an overall higher consumption of ASs/NNSs among those who were overweight or obese, whereas our findings on this were confounding. Again, this difference in findings may be due to the differences in the study population. Additionally, a 15-y longitudinal study in Norway assessing the changes in AS/NNS use from childhood to adulthood found a significant association between the use of ASs/NNSs and education level, with lower frequency of use among more highly educated individuals [47]. Bolt-Evensen et al. [47] found that participants consumed roughly 1.6 ASs/NNSs per week but did not provide the percentage of users compared with nonusers. Conversely, the present study found higher intake rates among more highly educated males and females; this may be due to the differences in the age of participants included in both the studies as Bolt-Evensen et al. [47] included both children and adults.

This review has some strengths that should be considered. First, to our knowledge, this is the first systematic review to assess the prevalence of AS/NNS use among females of reproductive age. Further, we searched an extensive range of databases (*n* = 9) and assessed the methodological quality of the included articles, ensuring that all possible research articles were included in our review. Our review included studies conducted in a range of HICs, thus including females from diverse cultures and backgrounds, enabling high generalizability of results within the context of HICs. Finally, we attempted to perform a meta-analysis of the pooled prevalence of the use of AS/NNS in the population.

We also faced some limitations in our review. There was high heterogeneity in the pooled prevalence (*I*^2^ = 99.9%); therefore, the results need to be cautiously interpreted. This high heterogeneity indicates that the combined effect size found in the meta-analysis may not be an accurate reflection of the true value in any specific study, leading to less precise estimates and higher risk of bias. The majority of the studies included both male and female participants; therefore, factors associated with NNS use among females were not separately reported. Only one [32] study reported the factors that impacted females of reproductive age and their use of ASs/NNSs. Due to this limitation, we had to interpret the results from both sexes; these factors need to also be interpreted cautiously. Further, all of the studies included in this review were cross-sectional studies with 33% high and 67% moderate quality.

Cross-sectional designs inherently limit the ability to infer causation, as exposure and outcome are measured simultaneously at only one time point, thus making it challenging to determine whether AS/NNS consumption is a cause or consequence of associated factors, such as BMI or income level. This further hinders our understanding of the duration of the relationship between ASs/NNSs and associated factors, as well as the long-term effects of AS/NNS use. Considering the World Bank country classifications by income level for 2024−2025, most of the included studies were conducted in HICs (*n* = 14). Only one study [34] that analyzed 4 countries included 2 countries, Peru and Guatemala, which are upper-middle-income countries. This limits the global generalizability of the results. We need to collect more evidence from LMICs and low-income countries (LICs) to better determine the global prevalence and factors associated with AS/NNS use among females of reproductive age.

The findings from our review highlight the urgent need for more high-quality studies that investigate the use of ASs/NNSs among females of reproductive age in LMICs/LICs. This lack of data on AS/NNS use in LMICs/LICs limits our abilities to appropriately acknowledge the risk factors that affect their use in these communities, thereby limiting the effectiveness of preventive and health promotion efforts aimed at reducing its use.

Furthermore, we need more studies that present data separately for males and females in this field to allow us to better understand the specific use, and factors for use, of ASs/NNSs between sexes to apply specific, and useful, preventive measures. This would also allow us to pool factors data to gain an insightful and in-depth understanding of the key drivers of AS/NNS use in females of reproductive age. Finally, more longitudinal studies are needed to better understand the effect that AS/NNS use during a female’s reproductive age has on their health during pregnancy as well as the health of their child. Future research could also benefit from longitudinal designs that track AS/NNS consumption and related health outcomes over time to better understand causal relationships.

The findings of this review have several implications for public health interventions aimed at managing AS/NNS consumption among females of reproductive age. Given the associations between AS/NNS use, socioeconomic factors, and lifestyle choices, tailored interventions that consider cultural, socioeconomic, and educational factors may be more effective in promoting informed dietary choices. Health education is an important, and highly useful, means of delivering AS/NNS information to females in diverse socioeconomic settings. Culturally sensitive and easy-to-digest online and in-person resources focusing on balanced nutrition choices should be made available to all females of reproductive age, enabling them to make informed decisions regarding their own nutritional decisions. Furthermore, future research should prioritize understanding the motivations behind AS/NNS use among females, particularly in relation to weight management, dietary flexibility, and health perceptions. Findings from these studies will be vital in codesigning effective educational resources and future interventions for a diverse range of females.

Moreover, investigating the impact of socioeconomic and cultural factors on AS/NNS consumption across different regions could help identify population-specific trends and barriers. In doing so, public health initiatives could be more precisely targeted to address the unique needs and behaviors of diverse demographic groups. Finally, given the high heterogeneity observed, further studies with more standardized methods and reporting protocols are recommended to enhance the comparability of findings across different populations.

In conclusion, our systematic review highlights the global prevalence and factors associated with AS consumption among nonpregnant, nonlactating females of reproductive age, providing a much-needed evidence base in this underresearched area of nutrition. The findings demonstrate a substantial variation in the prevalence of AS use across different populations, highlighting the influence of socioeconomic status, BMI, ethnicity, and lifestyle behaviors. Despite significant heterogeneity in study designs and reporting, our pooled data suggest a relatively high usage of ASs within this demographic, underscoring the importance of understanding motivations, perceived benefits, and potential health implications.

By addressing this gap in the literature, we aim to inform health care providers, policymakers, and females about the prevalence and associated factors of AS use. This research can support the development of targeted health education and interventions that empower females to make informed dietary choices during their reproductive years. Future studies are encouraged to explore the long-term health effects of AS consumption in this population to further guide evidence-based dietary recommendations.

## Author contributions

The authors’ responsibilities were as follows – ZSL, SJZ, MB, SSA: conceptualized the study; PC, KM, ZSL, SSA: contributed to screening, extraction, and quality assessment; SSA, ZSL: contributed to the analysis and generation of results; SSA, PC: wrote the first draft of the manuscript; and all authors: examined and contributed to the interpretation the results, provided guidance on methods, and read and approved the final manuscript.

## Funding

ZSL is supported by a National Health and Medical Research Council (NHMRC) Investigator Grant (#GNT2009730).

## Conflict of interest

The authors report no conflicts of interest.
