# Supplementary material for: Prevalence and Factors Associated with the Use of Artificial Sweeteners in Nonpregnant, Nonlactating Females of Reproductive Age – A Systematic Review
Source: Curr Dev Nutr. 2025 Jun 4;9(9):107478. doi: 10.1016/j.cdnut.2025.107478 (PMC12512156; doi:10.1016/j.cdnut.2025.107478)
Supplement: Multimedia component 1 [file mmc1.docx]

**Supplemental** **Appendix 1: Search terms and strategies**

Embase <1974 to **2024 May 08>**

1 Non-pregnant wom?n.tw. 7527

2 Non-lactating wom?n.tw. 247

3 Adult wom?n.tw. 13391

4 women.mp. 1692429

5 exp female/ 12017232

6 women of reproductive age.mp. or *adult/ 57011

7 woman of reproductive age.mp. 232

8 Adult female*.mp. 32345

9 reproductive age female*.mp. 283

10 (lady or ladies).mp. 17703

11 *girl/ 4031

12 *mother/ 24680

13 1 or 2 or 3 or 4 or 5 or 6 or 7 or 8 or 9 or 10 or 11 or 12 12257875

14 Frequen*.ti,ab. 2876647

15 Use*.ti,ab. 11731250

16 Consumption pattern*.ti,ab. 5295

17 Prevalence.ti,ab. 1191493

18 Consum*.ti,ab. 794988

19 determinant*.ti,ab. 360492

20 Risk factor*.ti,ab. 1182528

21 associated factor*.ti,ab. 53313

22 caus*.ti,ab. 4319849

23 Predictor.tw. 390100

24 indicator*.tw. 502904

25 influencer*.tw. 2524

26 exp Prevalence/ 1047119

27 exp "social determinants of health"/ 23247

28 exp "Health Status Indicators"/ 46211

29 ("Artificially sweetened" or "non nutritive sweet*" or saccharin or spartam* or stevia* or "artificial sweet*" or "diet drink*" or "diet beverage*" or "diet soda" or "diet cola" or "sugar twin" or "ultrasweet" or "sweet and low*" or "sugar substitute*" or splenda* or cyclamate* or "low joule sweetener*" or "Artificial sweeten*" or "Non nutritive sweeten*" or "Low calorie sweeten*" or "No calorie sweeten*" or "Non caloric sweeten*" or "Acesulfame potassium" or Sucralose or Neotame or Advantame or Xylitol or LNCS or "food additive*").tw. 24798

30 exp "Artificially Sweetened Beverages"/ 298

31 "Non Nutritive Sweeteners"/ 466

32 "Sweetening Agents"/ 6343

33 14 or 15 or 16 or 17 or 18 or 19 or 20 or 21 or 22 or 23 or 24 or 25 or 26 or 27 or 28 17951919

34 29 or 30 or 31 or 32 28589

35 13 and 33 and 34 4300

36 (exp animal/ or animal experiment.sh. or nonhuman.sh.) not ((exp animal/ or animal experiment.sh. or nonhuman.sh.) and (exp human/ or human experiment.sh.)) 7424775

37 35 not 36 3086

38 limit 37 to english language **2966**

**Pubmed 9/05/24**

Non-pregnant wom?n[tiab] OR Non-lactating wom?n[tiab] OR Adult wom?n[tiab] OR Adult human female*[tiab] OR Adult female*[tiab] OR Female*[tiab] OR Wom*n[tiab] OR Reproductive age wom?n[tiab] OR Reproductive age female*[tiab] OR Girl*[tiab] OR mother*[tiab] OR lady OR ladies OR "Female"[mh] OR "Women"[mh:noexp] AND Frequen*[tiab] OR Use[tiab] OR uses[tiab] OR "Consumption pattern*"[tiab] OR Prevalence[tiab] OR Consum*[tiab] OR Determinant* OR "Risk factor*" OR "associated factor*" OR caus* OR Predictor[tiab] OR indicator*[tiab] OR influencer*[tiab] OR Prevalence[mh] OR "Social Determinants of Health"[mh] OR "Health Status Indicators"[mh] AND "Artificially sweetened"[tiab] OR "non-nutritive sweet*"[tiab] OR Saccharin[Mesh:NoExp] OR saccharin[tiab] OR Aspartame[Mesh:NoExp] OR aspartam*[tiab] OR stevia[Mesh:NoExp] OR stevia[tiab] OR "artificial sweet*"[tiab] OR "diet drink*"[tiab] OR "diet beverage*"[tiab] OR "diet soda"[tiab] OR "diet cola"[tiab] OR "sugar twin" OR nutrasweet[tiab] OR " sweet and low*"[tiab] OR "sugar substitute*"[tiab] OR splenda*[tiab] OR cyclamates[Mesh] OR "low joule sweetener*"` OR "Artificial sweeten*"[tiab] OR "Non-nutritive sweeten*"[tiab] OR "Low-calorie sweeten*"[tiab] OR "No-calorie sweeten*"[tiab] OR "Non-caloric sweeten*"[tiab] OR "Acesulfame potassium"[tiab] OR Sucralose[tiab] OR Neotame[tiab] OR Advantame[tiab] OR Xylitol[tiab] OR LNCS[tiab] OR "food additive*"[tiab] OR "Artificially Sweetened Beverages"[Mesh] OR "Non-Nutritive Sweeteners"[Mesh] OR "Sweetening Agents"[Mesh]

Filter by human and english

Results: **7424**

**CINAHL**

Results 1,300

TI "Non-pregnant wom?n" OR AB "Non-pregnant wom?n") OR (TI "Non-lactating wom?n" OR AB "Non-lactating wom?n") OR (TI "Adult wom?n" OR AB "Adult wom?n") OR (TI "Adult human female*" OR AB "Adult human female*") OR (TI "Adult female*" OR AB "Adult female*") OR (TI Female* OR AB Female*) OR (TI Wom*n OR AB Wom*n) OR (TI "Reproductive age wom?n" OR AB "Reproductive age wom?n") OR (TI "Reproductive age female*" OR AB "Reproductive age female*") OR (TI Girl* OR AB Girl*) OR (TI mother* OR AB mother*) OR lady OR ladies OR (MH Female+) OR (MH Women)

AND

(TI Frequen* OR AB Frequen*) OR (TI Us* OR AB Us*) OR (TI "Consumption pattern*" OR AB "Consumption pattern*") OR (TI Prevalence OR AB prevalence) OR (TI Consum* OR AB Consum*) OR Determinant* OR "Risk factor*" OR "associated factor*" OR caus* OR (TI Predictor OR AB Predictor) OR (TI indicator* OR AB indicator*) OR (TI influencer* OR AB influencer*) OR (MH Prevalence+) OR (MH "Social Determinants of Health+") OR (MH "Health Status Indicators+")

AND

(TI "Artificially sweetened" OR AB "Artificially sweetened") OR (TI "non-nutritive sweet*" OR AB "non-nutritive sweet*") OR (MH Saccharin) OR (TI saccharin OR AB saccharin) OR (MH Aspartame) OR (TI aspartam* OR AB aspartam*) OR (MH stevia) OR (TI stevia OR AB stevia) OR (TI "artificial sweet*" OR AB "artificial sweet*") OR (TI "diet drink*" OR AB "diet drink*") OR (TI "diet beverage*" OR AB "diet beverage*") OR (TI "diet soda" OR AB "diet soda") OR (TI "diet cola" OR AB "diet cola") OR (TI "sugar twin" OR AB "sugar twin") OR (TI nutrasweet OR AB nutrasweet) OR (TI " sweet and low*" OR AB " sweet and low*") OR (TI "sugar substitute*" OR AB "sugar substitute*") OR (TI splenda* OR AB splenda*) OR (MH cyclamates+) OR (TI "low joule sweetener*" OR AB "low joule sweetener*") OR (TI "Artificial sweeten*" OR AB "Artificial sweeten*") OR (TI "Non-nutritive sweeten*" OR AB "Non-nutritive sweeten*") OR (TI "Low-calorie sweeten*" OR AB "Low-calorie sweeten*") OR (TI "No-calorie sweeten*" OR AB "No-calorie sweeten*") OR (TI "Non-caloric sweeten*" OR AB "Non-caloric sweeten*") OR (TI "Acesulfame potassium" OR AB "Acesulfame potassium") OR (TI Sucralose OR AB Sucralose) OR (TI Neotame OR AB Neotame) OR (TI Advantame OR AB Advantame) OR (TI Xylitol OR AB Xylitol) OR (TI LNCS OR AB LNCS) OR (TI "food additive*" OR AB "food additive*") OR (MH "Artificially Sweetened Beverages+") OR (MH "Non-Nutritive Sweeteners+") OR (MH "Sweetening Agents+")

Filter by English and humans

**CENTRAL/Cochrane**

Search Name:

Date Run: 09/05/2024 06:49:57

Comment:

ID Search Hits

#1 ("Non-pregnant" NEXT wom?n):ti,ab 819

#2 ("Non-lactating" NEXT wom?n):ti,ab 82

#3 ("Adult" NEXT wom?n):ti,ab 1721

#4 ("Adult human" NEXT female*):ti,ab 2

#5 ("Adult" NEXT female*):ti,ab 1280

#6 Female*:ti,ab 126870

#7 Wom*n:ti,ab 192866

#8 ("Reproductive age" NEXT wom?n):ti,ab 258

#9 ("Reproductive age" NEXT female*):ti,ab 13

#10 Girl*:ti,ab 9440

#11 mother*:ti,ab 24262

#12 lady or ladies 899

#13 MeSH descriptor: [Female] explode all trees 603955

#14 MeSH descriptor: [Women] explode all trees 1489

#15 #1 or #2 or #3 or #4 or #5 or #6 or #7 or #8 or #9 or #10 or #11 or #12 or #13 or #14 787525

#16 Frequen*:ti,ab 172654

#17 Use:ti,ab or usage* or use* 2159005

#18 ("Consumption" NEXT pattern*) 202

#19 Prevalence:ti,ab 41955

#20 Consum*:ti,ab 83236

#21 Determinant*:ti,ab 10133

#22 ("Risk" NEXT factor*):ti,ab 53945

#23 ("associated"):ti,ab 305798

#24 associated NEXT factor*:ti,ab 925

#25 caus*:ti,ab 158891

#26 Predictor:ti,ab 18079

#27 indicator*:ti,ab 24658

#28 influencer*:ti,ab 106

#29 MeSH descriptor: [Prevalence] 3 tree(s) exploded 7402

#30 MeSH descriptor: [Social Determinants of Health] 1 tree(s) exploded 81

#31 MeSH descriptor: [Health Status Indicators] 2 tree(s) exploded 29518

#32 #16 or #17 or #18 or #19 or #20 or #21 or #22 or #23 or #24 or #25 or #26 or #27 or #28 or #29 or #30 or #31 2159064

#33 "Artificially sweetened":ti,ab 123

#34 ("non-nutritive" NEXT sweet*):ti,ab 100

#35 saccharin:ti,ab 253

#36 aspartam*:ti,ab 259

#37 stevia:ti,ab 131

#38 ("artificial" NEXT sweet*):ti,ab 186

#39 ("diet" NEXT drink*):ti,ab 32

#40 ("diet" NEXT beverage*):ti,ab 40

#41 "diet soda":ti,ab 34

#42 "diet cola":ti,ab 26

#43 "sugar twin":ti,ab 0

#44 nutrasweet:ti,ab 3

#45 ("sweet and" NEXT low*):ti,ab 2

#46 ("sugar" NEXT substitute*):ti,ab 65

#47 splenda*:ti,ab 22

#48 ("low joule" NEXT sweetener*):ti,ab 0

#49 ("Artificial" NEXT sweeten*):ti,ab 186

#50 ("Non-nutritive" NEXT sweeten*):ti,ab 99

#51 ("Low-calorie" NEXT sweeten*):ti,ab 50

#52 ("No-calorie" NEXT sweeten*):ti,ab 3

#53 ("Non-caloric" NEXT sweeten*):ti,ab 40

#54 "Acesulfame potassium":ti,ab 18

#55 Sucralose:ti,ab 286

#56 Neotame:ti,ab 5

#57 Advantame:ti,ab 1

#58 Xylitol:ti,ab 739

#59 LNCS:ti,ab 9

#60 ("food" NEXT additive*):ti,ab 212

#61 MeSH descriptor: [Saccharin] explode all trees 69

#62 MeSH descriptor: [Aspartame] explode all trees 128

#63 MeSH descriptor: [Stevia] explode all trees 25

#64 MeSH descriptor: [Artificially Sweetened Beverages] explode all trees 9

#65 MeSH descriptor: [Non-Nutritive Sweeteners] explode all trees 57

#66 MeSH descriptor: [Sweetening Agents] explode all trees 954

#67 #33 or #34 or #35 or #36 or #37 or #38 or #39 or #40 or #41 or #42 or #43 or #44 or #45 or #46 or #47 or #48 or #49 or #50 or #51 or #52 or #53 or #54 or #55 or #56 or #57 or #58 or #59 or #60 or #61 or #62 or #63 or #64 or #65 or #66 2823

#68 #15 and #32 and #67 1392

Web of Science

Results: 1,302 (English filter)

AB: "Non-pregnant wom?n" OR "Non-lactating wom?n" OR "Adult wom?n" OR "Adult human femal*" OR "Adult femal*" OR Femal* OR Wom?n OR "Reproductive age wom?n" OR "Reproductive age female*" OR Girl* OR mother* OR lady OR ladies OR Female OR Women

AND

AB: Frequen* OR Use OR Usage* OR uses OR “Consumption pattern*" OR prevalence OR Consum* OR Determinant* OR "Risk factor*" OR "associated factor*" OR caus* OR Predictor OR indicator* OR influencer* OR Prevalence OR "Social Determinants of Health" OR "Health Status Indicators"

AND

AB: "Artificially sweetened" OR "non-nutritive sweet*" OR saccharin OR aspartam* OR stevia OR "artificial sweet*" OR "diet drink*" OR "diet beverage*" OR "diet soda" OR "diet cola" OR "sugar twin" OR nutrasweet OR "sweet and low*" OR "sugar substitute*" OR splenda* OR cyclamates OR "low joule sweetener*" OR "Artificial sweeten*" OR "Non-nutritive sweeten*" OR "Low-calorie sweeten*" OR "No calorie sweeten*" OR "Non caloric sweeten*" OR "Acesulfame potassium" OR Sucralose OR Neotame OR Advantame OR Xylitol OR LNCS OR "food additive*" OR "Artificially Sweetened Beverages" OR "Non-Nutritive Sweeteners" OR "Sweetening Agents"
